# The Initial Experience With Core Decompression and Reversed Autologous Bone Grafting for Post-COVID-19 Avascular Necrosis of the Femoral Head in Central India

**DOI:** 10.7759/cureus.102454

**Published:** 2026-01-28

**Authors:** Anil V Golhar, Sarang N Rokade, Manish S Kawade, Sushil H Mankar, Abhishek C Golhar, Vijay M Kane

**Affiliations:** 1 Orthopaedics and Trauma, N. K. P. Salve Institute of Medical Sciences & Research Centre and Lata Mangeshkar Hospital, Nagpur, IND; 2 Orthopaedics, Government Medical College and Hospital, Nagpur, Nagpur, IND; 3 Joint Reconstructions-Revision Hip and Knee Koints, George Eliot Hospital Trust, Nuneaton, GBR

**Keywords:** autogenous bone graft, avascular necrosis: avn, core-decompression, hip joint, reverse bone graft

## Abstract

Avascular necrosis (AVN) of the femoral head has emerged as a significant complication following the coronavirus disease 2019 (COVID-19) pandemic, affecting a relatively young patient population. Core decompression with reverse autologous bone grafting is a key joint-preserving surgical technique. This technical report describes our single-center experience and illustrates the application of this technique in a series of post-COVID-19 AVN cases.

This retrospective technical assessment was conducted at a single center in Nagpur, central India, spanning from January 2022 to December 2023. The study included 101 hips (55 patients) who underwent the core decompression procedure utilizing reversed autologous bone grafting. The report describes the operative steps, specific surgical pearls, and technical challenges encountered during this unique approach in post-COVID-19 AVN patients.

The procedure demonstrated favorable short-term outcomes, with patients showing statistically significant postoperative improvements in pain, functional mobility, gait stability, and overall mobility, as measured by Harris Hip Scores (p<0.0001). The technique of core decompression with reversed bone grafting is a viable and effective strategy for treating AVN of the femoral head in the post-COVID-19 era. This report offers practical insights into the application and efficacy of the technique, serving as a procedural guide for other orthopedic centers.

## Introduction

The global health landscape has been significantly altered by the coronavirus disease 2019 (COVID-19) pandemic, leaving a spectrum of systemic complications in its wake, including those affecting the skeletal system [1,2,3]. Avascular necrosis (AVN) of the femoral head has emerged as a major post-COVID-19 complication. It is primarily observed in patients who tested positive for COVID-19 via reverse transcription polymerase chain reaction (RT-PCR) and is often strongly associated with the subsequent use of corticosteroid therapy during their acute illness [4,5,6]. This condition predominantly affects younger patients and, if not managed promptly, can progress to femoral head collapse, resulting in severe hip joint osteoarthritis that ultimately requires arthroplasty.

The pandemic has been associated with a well-documented increase in the incidence of AVN [7]. A similar upward trend has been observed among younger individuals (aged 20-40 years) in central India (Vidarbha region), with a notable male predominance. Early surgical intervention is essential to prevent femoral head collapse and preserve the native joint. Other modalities, such as vascularized bone grafts and rotational osteotomies, require high technical expertise, specialized instrumentation, and are associated with variable rates of complications and high expenditure [8-13].

Core decompression with modifications remains a safe, widely practiced, and evidence-based procedure for the pre-collapse stage of femoral head AVN. This technical report describes our initial experience, outlines the precise operative steps, and summarizes short-term outcomes of core decompression combined with reverse autologous bone grafting performed at our center for the management of post-COVID-19 AVN of the femoral head in this patient cohort.

## Technical report

Materials and methods

Study Design and Ethics

We conducted a single-center retrospective chart review and technical assessment at our institute in Nagpur, central India. The study population included cases treated from January 1, 2022, to December 31, 2023. Ethical clearance for this retrospective analysis was obtained from the Institutional Ethics Committee (NKP Salve Institute of Medical Sciences and Research Centre and Lata Mangeshkar Hospital) on May 22, 2025 (approval number: LMH/IEC/13/2025). All procedures involving human participants adhered to the ethical standards of the institutional and national research committees and the 1964 Declaration of Helsinki, including its later amendments. Proper written informed consent was obtained from the patients and their relatives for the procedure at the time of the index surgery, and additional consent was obtained for the publication of study data.

Patient Selection and Exclusion Criteria

Inclusuion criteria: Out of 80 patients treated with core decompression and reversed autologous bone grafting during this period, 55 patients (101 hips) were included in the final analysis, as they met the following criteria: a diagnosis of AVN Grade I, Grade II A, or Grade II B; completion of a minimum of one year of postoperative follow-up; and a confirmed history of COVID-19 infection via RT-PCR. Patients were evaluated for a history of steroid use during their COVID-19 illness; however, given the chaotic state of the local health system during the pandemic, comprehensive records were often unavailable. Inclusion was not strictly limited to steroid-treated patients; the cohort included both patients with a confirmed history of steroid use and those who were RT-PCR-positive but asymptomatic/mild and did not receive steroid therapy.

Exclusion criteria: Patients with AVN secondary to other etiologies, such as Sickle Cell Disease (as they are prone to recurrent vaso-occlusive crises that can nullify the principle of core decompression and are prevalent in this area of India [14]), patients who were on steroids or received prolonged steroids for some other diseases and patients who had not completed a minimum of one year of follow-up were excluded.

Preoperative Evaluation

Patients meeting the inclusion criteria were evaluated for age, sex, occupation, pain, limp, symptom duration, progression, deformity, and history of other joint pain. Risk factors, specifically alcoholism and steroid administration during COVID-19, were noted. Investigations included routine blood tests, COVID-19 screening, plain radiographs (anteroposterior and frog leg views X-RAYS), and MRI of the pelvis to confirm the stage of the disease and status of the contralateral hip.

Instrumentation

Our method utilizes basic, simple, and easily reproducible instrumentation, which is locally made, cost-effective, and designed for re-use. No adjuvant therapy was used in this study cohort.

Surgical technique

All surgeries were performed by the same surgical team.

A. Anesthesia and Positioning

The surgery was performed under spinal anesthesia, with general anesthesia used only if strongly requested by the patient. The patient was positioned supine on a fracture table, and the perineal post was centered to allow for simultaneous access to both hips. The limb to be operated on was kept straight and slightly internally rotated to align the femoral neck horizontally. Sterile painting and draping were performed bilaterally.

B. Guide Wire Placement, Decoring, and Decompression

**Figure 1:**
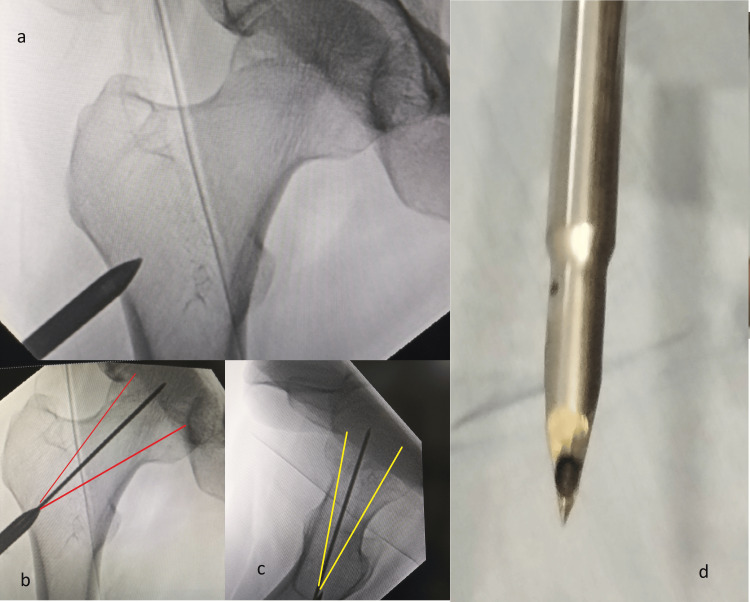
Trochar insertion and guide wire placement The cannulated trochar (d), which can be made from a cannulated screw driver or drill bit, is put over the desired entry point over the trochanteric region under image intensifier guidance and inserted by slight tapping or applying rotational force (a). Through this trochar the guide wire is placed in the desired sector in anteroposterior and lateral views (b, c)

The incision site was marked under fluoroscopy guidance. A 2-3 cm incision was made at the trochanteric ridge. A sharp cannulated trocar was used to make an entry point into the lateral femur. This ensures the safe placement of the guide wire into the desired sector of the femoral head (Figure 1). The guide wire placement was confirmed using fluoroscopy in anteroposterior and lateral views.

**Figure 2:**
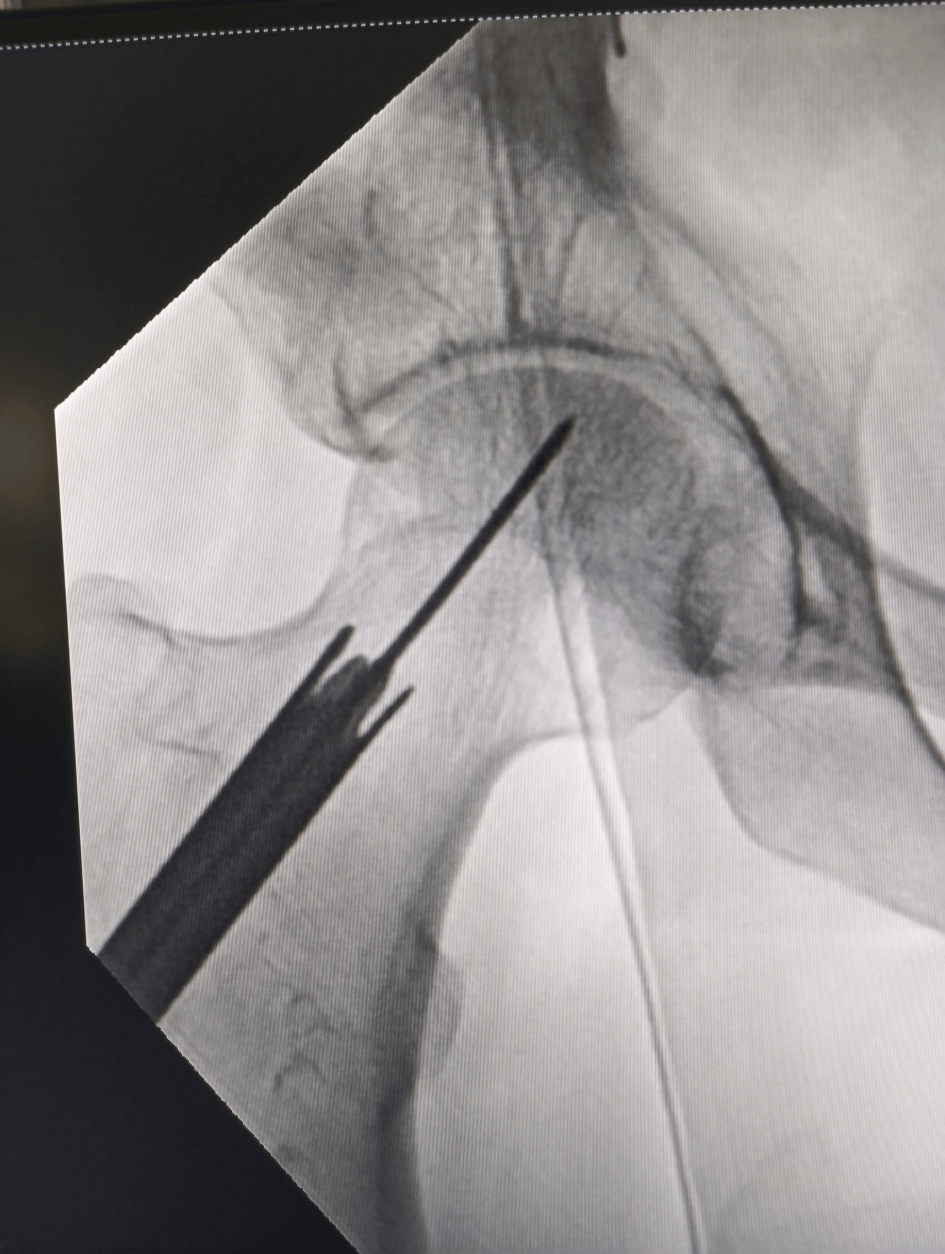
Decoring The decoring or a core removal is done with a hollow mill inserted under image intensifier guidance till the desired area with clockwise and anticlockwise movement

A specialized, hollow-mill-like instrument with a canal diameter of 10, 11, or 12 mm was used to decore the femoral head under C-arm guidance (Figure 2).

C. Reversed Autologous Bone Grafting

**Figure 3:**
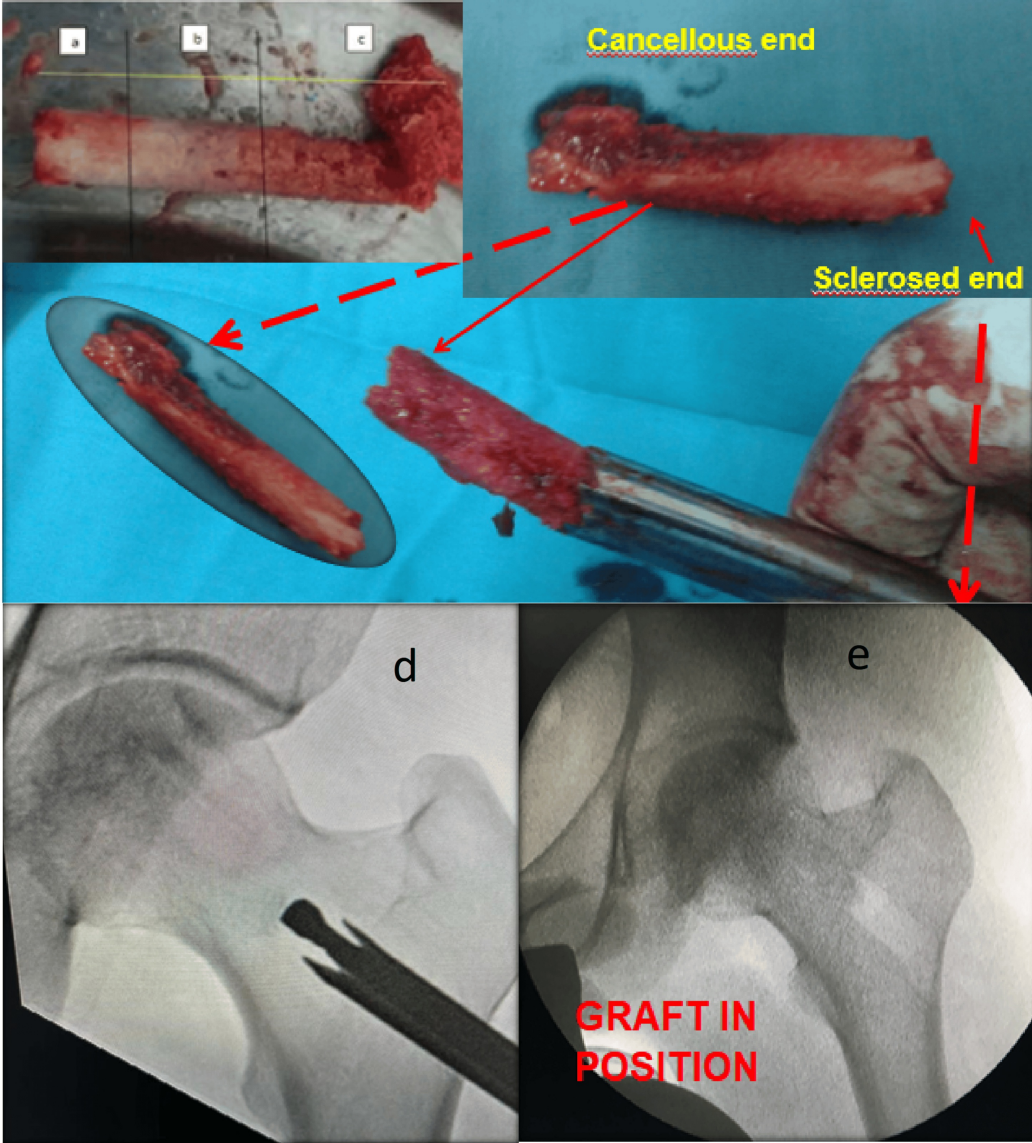
Reversal of the graft The core retrieved from the femoral head reversed generally shown the whitish sclerotic (a), mixed (b) and good cancellous zone (c). This graft is reversed and sclerotic bone side stuffed inside the hollw mill first and hollow mill put inside the decored area under image intensifier guidance. the graft is hammered back inside the canal (d, e)

As the decoring/hollow mill instrument advances, the bone sleeve typically breaks automatically at the interface between the normal/edematous bone and the sclerotic/hard bone. This bone sleeve, or procured bone graft, comes out into the hollow of the instrument. The sclerotic bone at the site of the AVN was then drilled over the guide wire in the desired locations under fluoroscopy guidance. The procured bone graft was then reversed and stuffed into the canal. The trochanteric part (red-red zone) was placed toward the femoral head side, and the head side (white-white zone) was placed toward the trochanter (Figure 3).

[Surgeon’s note: At this step, the bone graft can be soaked with platelet-rich plasma, bone marrow aspirate, or zoledronic acid as per the surgeon’s preference. This step was omitted in the present study.]

The decoring instrument was then put back into the canal prepared in the femoral head, and the reversed bone graft was pushed into the head with the help of a plunger to stuff the femoral head under fluoroscopy control. It is not necessary to fill the trochanteric void, as it is known to heal spontaneously. Closure was done in layers.

D. Postoperative Management and Salvage Cases

Patients were advised to follow strict non-weight-bearing precautions for a minimum of six weeks. Follow-up evaluations with X-rays were performed at one, three, and six months, and at one and two years postoperatively. Two patients with Grade IV AVN on one side and three on the other underwent total hip replacement (THR) for the Grade IV hip while simultaneously receiving core decompression with bone grafting on the contralateral hip. Additional grafting material was harvested from the excised femoral heads as a salvage procedure, with patients providing informed consent regarding the potential future need for THR on the grafted hip.

Results

This study included 55 patients (101 hips) who fulfilled the criterion of a minimum one-year follow-up and provided a Harris Hip Score (HHS).

Demographics and Preoperative Staging

The majority of the patient cohort were males (90.1%), with a higher proportion of bilateral presentations (80.2%). The primary age groups affected were 31-40 years (33.7%) and 20-30 years (30.7%) (Table 1).

**Table 1:** Demographic profile of patients SD: standard deviation

		No. of patients (n=101)	Percenatge
Age group, years	20-30	31	30.7
	31-40	34	33.7
	41-50	18	17.8
	51-60	13	12.9
	>60	05	4.9
	Mean ± SD		
Gender	Male	91	90.1
	Female	10	9.9
Laterality	Right	13	12.9
	Left	07	6.9
	Bilateral	81	80.2

Table 2 provides information about the staging of patients both at baseline and at their final follow-up. Initially, the majority of hips were in Stage I (54.4%). At the final follow-up, disease progression was observed in some patients, as shown by a slight increase in Stage III and the appearance of Stage IV (1.9%) in a small percentage. The rationale for performing decoring and reversed grafting in patients initially classified as Stage III was to reduce pain and delay the need for THR in the hip contralateral to a Stage IV hip.

**Table 2:** Stage at presentation and at final follow-up

		No. of patients (n=101)	Percenatge
Stage	I	55	54.4
	II	39	38.6
	III	7	6.9
Stage at final follow-up	I	50	49.5
	II	40	39.6
	III	9	8.9
	IV	2	1.9

Functional Outcomes (Harris Hip Score)

The preoperative and postoperative HHS were compared using a paired t-test (SPSS Statistics version 25). The overall analysis showed a statistically significant improvement in the total HHS postoperatively (p<0.0001), with an average increase of 9.59 points across all domains (Table 3).

**Table 3:** Comparison of the mean differences in parameters between preoperative and postoperative periods The mean total HHS score improved significantly by 9.59 points at the final follow-up (p<0.0001) HHS: Harris Hip Score; SD: standard deviation; ROM: range of motion; S: statistically significant; NS: not statistically significant

HHS		Mean	SD	Mean difference	t-value	p-value
Pain	Preoperative	26.63	5.70	6.01	9.23	<0.0001 (S)
	Postoperative	32.65	6.38			
Limp	Preoperative	9.27	1.71	1.31	6.14	<0.0001 (S)
	Postoperative	10.58	1.34			
Support	Preoperative	9.97	1.88	0.58	2.88	0.005 (S)
	Postoperative	10.55	1.43			
Distance walked	Preoperative	9.06	2.26	0.95	4.90	<0.0001 (S)
	Postoperative	10.01	1.70			
Sitting	Preoperative	4.34	0.94	0.45	4.34	<0.0001 (S)
	Postoperative	4.80	0.60			
Enter public transportation	Preoperative	1.00	0.00	00	--	--
	Postoperative	1.00	0.00			
Stairs	Preoperative	3.04	1.00	0.67	7.12	<0.0001 (S)
	Postoperative	3.72	0.69			
Shoes/socks	Preoperative	3.42	0.90	0.47	5.58	<0.0001 (S)
	Postoperative	3.90	0.44			
Deformity	Preoperative	4.00	0.00	00	-	-
	Postoperative	4.00	0.00			
ROM	Preoperative	4.28	0.27	0.11	0.928	0.356 (NS)
	Postoperative	4.39	1.17			
Total	Preoperative	73.57	12.44	9.59	7.76	<0.0001 (S)
	Postoperative	83.16	12.41			

Statistically significant improvements (p<0.0001) were noted across key domains: pain relief: average increase of 6.01 points; limp score: average increase of 1.31 points (better gait stability); distance walked: average increase of 0.95 points (improved mobility); significant improvements in sitting comfort, stair climbing, and ease of putting on shoes/socks.

Variables such as deformity, range of motion (ROM), and entering public transportation showed no statistically significant change (p>0.05).

## Discussion

Etiology and patient cohort

The demographic findings align with existing studies, showing a predominance of middle-aged males (30-50 years) in the affected cohort [15]. The post-COVID-19 surge of complications, often termed “Long COVID-19,” has been noted globally [16,17,18]. Our study exclusively included patients with a confirmed history of COVID-19 infection (RT-PCR positive) and observed the widespread use of steroids during their treatment [19]. The difficulty in retrospectively confirming steroid administration in all patients is a known limitation of data collected during the COVID-19 pandemic. Our local observation from central India of AVN involving bones beyond the femoral head further emphasizes the systemic impact.

Technical rationale and outcomes

The ultimate surgical aim is to achieve a painless, mobile hip, necessitating the preservation of femoral head sphericity [11,13]. Our procedure utilizes core decompression combined with reversed autologous bone grafting, a simple technique aiming to achieve localized structural support and biological stimulation. The primary benefit is the use of autologous cancellous graft harvested directly from the femoral neck/greater trochanter, eliminating the morbidity and cost associated with iliac crest harvesting or using expensive fixation devices [14]. Reversing the graft (trochanteric bone toward the head) ensures that structurally superior cancellous bone is packed into the necrotic cavity.

Addressing technical challenges

The bone quality harvested varies with the stage of AVN, from soft in early stages (oedema) to hard in sclerotic stages. The bone sleeve obtained sometimes shows three distinct zones (sclerotic, mixed, and normal), visually correlating to the underlying pathology (Figure 3). We consider the autograft harvested from the core to be sufficient to fill the cavity created, contradicting some authors who suggest insufficiency [10]. Using the autograft is a cost-effective and practical solution compared to synthetic substitutes or allografts for resource-limited settings like ours.

Comparison and conclusion

Our technique is minimally invasive and simple compared to procedures such as osteotomies, tantalum rods, or vascular grafts, which require more expertise and can alter local anatomy, complicating future total arthroplasty [20-26]. The procedure's basic instrumentation and ease of reproducibility make it feasible for centers with limited resources.

## Conclusions

Decoring and reversed autologous bone grafting is a safe, easily reproducible, and cost-effective surgical procedure. It demonstrated good clinical and functional outcomes without requiring adjunct therapies in pre-collapse AVN. We recommend this procedure as a viable, less invasive option that can be performed at various levels of surgical centers.
